# Acute COVID-19 Cerebellitis: A Rare Neurological Manifestation of COVID-19 Infection

**DOI:** 10.7759/cureus.18505

**Published:** 2021-10-05

**Authors:** Srikrishna V Malayala, Praveena Jaidev, Rachana Vanaparthy, Taranjeet S Jolly

**Affiliations:** 1 Internal Medicine, Temple University Hospital, Philadelphia, USA; 2 Internal Medicine, University of Wisconsin, Madison, USA; 3 Research, Physicians for American Healthcare Access, Philadelphia, USA; 4 Psychiatry and Behavioral Sciences, Penn State Health Milton S. Hershey Medical Center, Hershey, USA

**Keywords:** stroke and covid-19, covid-19 neurological outcomes, corticosteroids in covid-19, covid-19-related encephalopathy, white matter changes on mri, covid 19

## Abstract

The COVID-19 virus is well known to cause respiratory tract infections but several non-respiratory presentations and post-infectious complications have been well reported since its origin.

With this case report, we present a rare manifestation of COVID-19 infection that presented as acute cerebellitis. This is a case of a 63-year-old Caucasian male patient who presented with altered mental status and ataxia. He was diagnosed with COVID-19 infection about five days prior to presenting to the hospital. Neurological exam was consistent with cerebellar symptoms like broad bases gait, truncal ataxia and subsequent imaging revealed white matter degeneration and edema of the cerebellar hemispheres. The symptoms completely resolved following treatment of ongoing COVID-19 infection with corticosteroids and intravenous remdesivir.

With this case report, we intend to discuss the available literature regarding the clinical manifestations, management, and prognosis of COVID-19-induced cerebellitis.

## Introduction

Acute cerebellitis or acute cerebellar ataxia is an inflammatory syndrome characterized by acute onset of cerebellar signs and symptoms such as ataxia, nystagmus, and dysmetria. Acute viral cerebellitis is associated with fever, nausea, headache, changes in mental status and imaging often shows white matter degenerative changes in the cerebellum [1]. Post viral cerebellitis is relatively rare in adults with the majority of cases described in children younger than 3 years [2]. Since the onset of COVID-19 pandemic, a significant number of cases of COVID-19-induced neurological manifestations have been reported, especially in elderly patients with other comorbidities [3-6]. Majority of the cases reported have been within a few days to weeks following COVID-19 infection but sometimes this could be the initial presentation of COVID-19 without associated respiratory symptoms [7]. In Wuhan, China, 36.4% of COVID-19 positive patients were found to have some neurological manifestations [8]. Therefore, it is imperative for physicians to consider this COVID-19 cerebellitis in the differential diagnosis when the patient is presenting with fever and cerebellar symptoms. In this case report, we present the clinical presentation of a COVID-19-induced cerebellitis in a 63-year-old unvaccinated male patient and discuss the associated literature.

## Case presentation

A 63-year-old male with a past medical history of asthma, hypertension, paroxysmal atrial fibrillation (on Apixaban for anticoagulation), hypothyroidism, rheumatoid arthritis (on methotrexate), morbid obesity, and obstructive sleep apnea was brought into the emergency room for evaluation of confusion two days prior to admission to the hospital. Prior to arrival at our facility, he was seen at a different hospital five days ago for symptoms of low-grade fever, chills, fatigue, and nonproductive cough. He tested positive for COVID-19 infection via PCR. His infection was considered mild, he was managed conservatively and discharged after one day of hospitalization. After discharge, he continued to have persistent fevers and chills. His family noticed him to be confused and brought him back to the emergency room for re-evaluation. On initial evaluation, the patient was hypoxic and febrile with a temperature of 101.3 F. A complete review of systems could not be performed due to his confusion. He was found to be oriented only to self but not oriented to time and place on the mental status examination. Pupils were equal and reactive and no cranial nerve deformity was identified. He was able to follow some commands and was able to move all the extremities without any focal weakness. The sensory system was suboptimal, owing to his mental status. Auscultation of the lungs had scattered crackles in all the lung fields. The abdominal exam was benign with no tenderness or signs of fluid accumulation.

**Figure 1:**
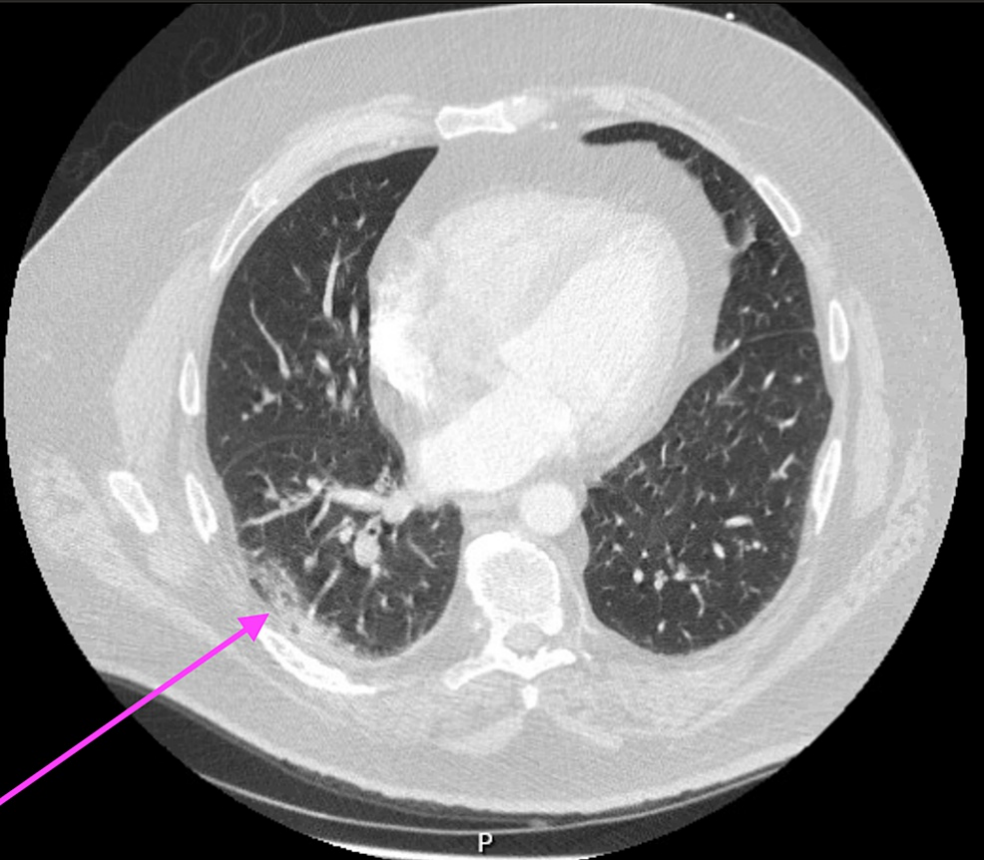
Peripheral ground-glass infiltrates in the lungs from COVID-19 pneumonia

Initial laboratory data showed elevated inflammatory markers with a C-reactive protein level of 7.7 mg/dl (normal range 0-0.8 mg/dl), fibrinogen level of 623 mg/dl (normal range 90-480 mg/dl), and lactate dehydrogenase (LDH) level 343 U/l (reference range 98-192 U/l). Otherwise, the electrolyte panel, renal function tests, and hepatic function panel were within normal limits. Chest X-ray did not show any abnormality and a contrast-enhanced CT scan showed subpleural ground-glass infiltrates in the right lower lobe, left upper and lobes, features consistent with COVID-19 pneumonia (Figure 1). A CT scan of the head was done to evaluate the change in mental status. It did not reveal any gross acute intracranial abnormality.

He was admitted to the floor with a preliminary differential diagnosis of delirium in the setting of ongoing hypoxia and fever from the COVID-19 infection. Treatment was initiated with systemic steroids, intravenous remdesivir, bronchodilators and anti-pyretics. The ongoing infection also triggered the atrial fibrillation and he went into a phase of rapid ventricular response. This was corrected by adjusting the doses of his home medications flecainide and metoprolol.

**Figure 2:**
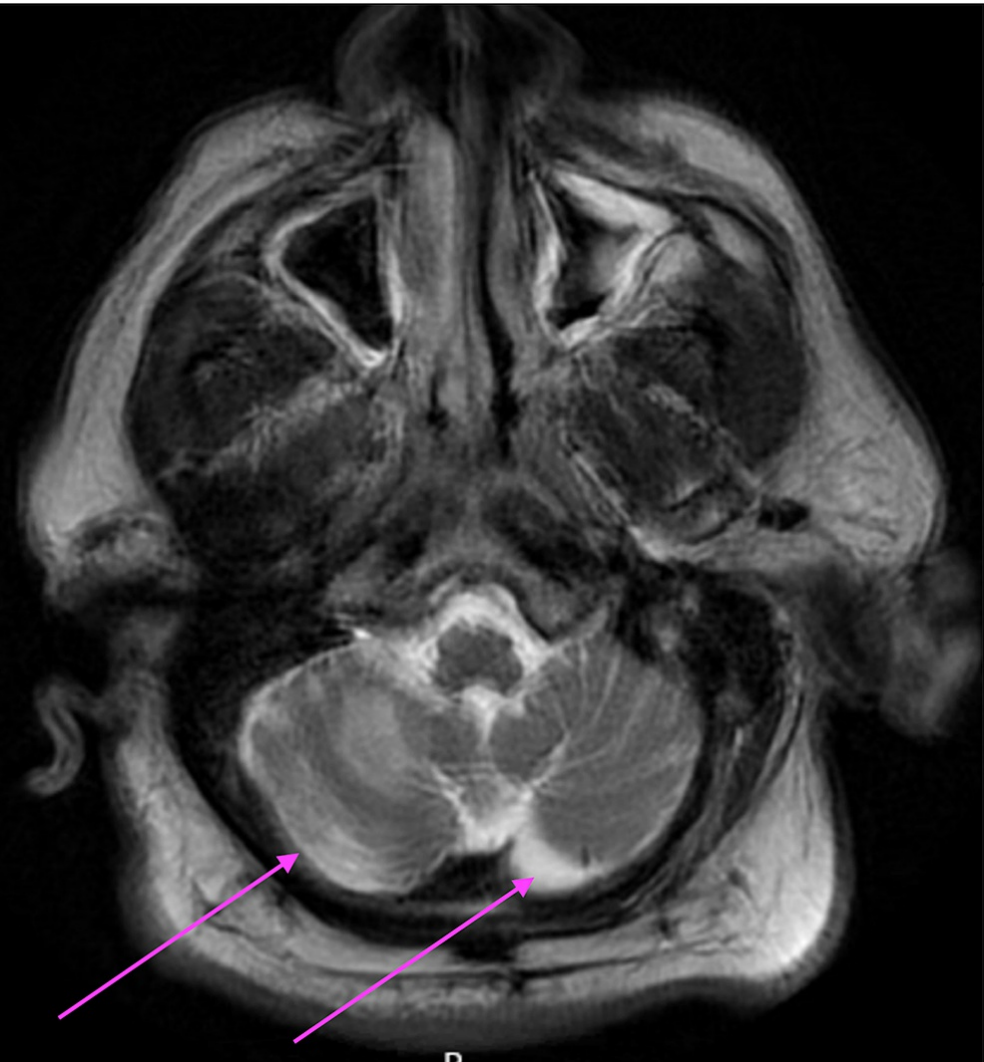
MRI brain showing bilateral cerebellar white matter signal abnormalities in COVID-19 cerebellitis

His fever and respiratory status improved over the next three days but there was no improvement in his mental status. He also gradually started having oral dyskinesias, which were not noted earlier. A comprehensive neurological exam also showed truncal ataxia and a broad-based gait. Otherwise, there was no other focal neurological defect noted. Further review of the history did not reveal any psychiatric history or co-morbidities and he was never on any antipsychotics. Differential diagnoses considered were drug-induced dyskinesias (metoclopramide was administered to treat nausea on a few occasions), an acute cerebrovascular accident (CVA), and COVID-19 cerebellitis. MRI of the brain without contrast enhancement was done at this point to evaluate the mental status and dyskinesis. It showed bilateral cerebellar white matter signal abnormalities extending to bilateral cerebellar peduncles (Figure 2).

**Figure 3:**
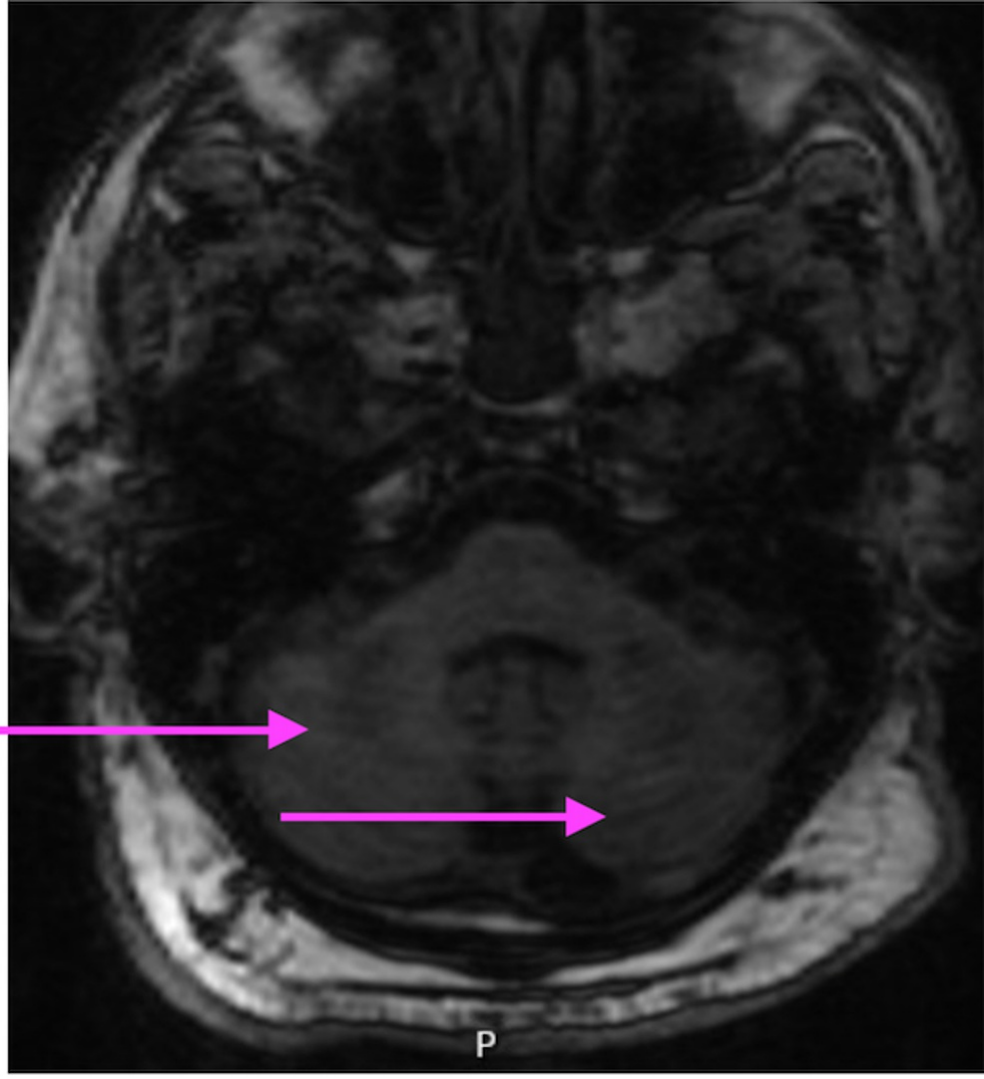
COVID-19-induced inflammation in the bilateral anterior inferior cerebellar artery territories

To further delineate the cerebellar lesions, MRI of the brain was performed with and without contrast which revealed bilateral brachium pontis lesions implying persistent inflammation in the bilateral anterior inferior cerebellar artery territories (Figure 3).

Based on the clinical presentation, imaging findings, and available literature, COVID-19 cerebellitis was strongly suspected. All the offending agents that could cause dyskinesias were discontinued. The steroids and remdesivir were continued for managing the COVID-19 pneumonia and cerebellitis. The symptoms gradually improved over the next four days and he was transitioned to a skilled nursing facility.

## Discussion

It is now known that COVID-19 infection has a wide range of clinical presentations including neurological symptoms. Even in very early reports from Wuhan, China in March/April 2020, neurological manifestations from COVID-19 infection were reported, more often in critically ill patients [8]. It was later noticed that many patients now have residual neurological and cognitive symptoms even after an extended period of time, also termed as COVID-19 long haulers [9].

The virus may gain access to the central nervous system either through the hematogenous route or through the olfactory bulb. The possible etiopathogenic mechanisms responsible for the neurological involvement are direct neuronal injury, immune-mediated injury, and injury secondary to hypoxemia [10]. Most of the studies report neurological involvement in 20-40% of patients hospitalized due to COVID-19 virus infection, mostly in the form of encephalopathy, meningitis, acute disseminated encephalomyelitis, and strokes [11].

Acute cerebellitis is a rare inflammatory syndrome usually presented with fever, nausea, headache, altered mental status along with acute onset of cerebellar signs and symptoms such as ataxia, nystagmus, or dysmetria [12]. This disease presentation is often caused due to viruses including varicella zoster, chickenpox, herpes simplex, rotavirus, Epstein-Barr virus (EBV) or cytomegalovirus (CMV), etc. and rarely caused due to medications including anti-epileptics, antineoplastics, lithium, or benzodiazepines [13]. Fadakar et al. described the first case of acute cerebellitis from COVID-19 infection [7]. Cerebellar ataxia occurring post-COVID-19 infection has been rarely reported in literature further adding to the gamut of neurological involvement of COVID-19 infection [7,14].

As ataxia in our patient occurred after COVID-19 infection had resolved and cerebrospinal fluid (CSF) was normal, the cerebellar involvement was possibly immune-mediated in nature. The rapid response with steroid pulse therapy further strengthens this hypothesis.

The patient in our case report was immunosuppressed as he was taking methotrexate for rheumatoid arthritis. We could not identify any literature that could explain the severity of COVID-19 cerebellitis or any other neurological presentation in immunosuppressed patients. We also cannot comment on the correlation between the severity of COVID-19 infection and the incidence of cerebellitis. The patient in our case report did not have a severe COVID-19 pneumonia as such but still had an atypical presentation with the cerebellar involvement.

The differential diagnosis for acute cerebellitis includes stroke, infectious meningoencephalitis, cerebellar tumors, acute disseminated encephalomyelitis, and posterior reversible encephalopathy syndrome. As the presentation is similar, it is very important to rule out other etiologies. MRI is the gold standard test for the diagnosis of cerebellitis showing cortical hypointensity or hyperintensity, swelling, cortical or leptomeningeal enhancement [14]. Cerebrospinal fluid (CSF) analysis shows increased protein and lymphocytosis. However, confirmation with CSF COVID-19 RT-PCR was not done in this case as it was not available in our facility [15]. This is a limitation of this case report.

In the era of COVID-19 variants and the new mutations of the virus, there is always a potential of atypical presentations of the COVID-19 infection [16,17]. The hesitancy around taking the widely available COVID-19 vaccinations has also contributed to the recurrent and ongoing waves of the pandemic [18-20]. It is also prudent to be aware of medication interactions, especially the psychotropic medications used for the management of delirium in the backdrop of medications used to treat COVID-19 infection. These patients should also be closely followed up in the ambulatory setting to ensure the resolution of the symptoms.

## Conclusions

As this global pandemic has surrounded the humanity on all sides and varied neurologic and other manifestations of this infection are being recognized, physicians should remain updated and should not ignore any of the atypical clinical presentations from COVID-19. In this particular case report, we identified a patient who presented with dysmetria and was diagnosed with COVID-19 cerebellitis. The diagnosis was based on clinical presentation and imaging. The management of the cerebellar symptoms in our case was systemic corticosteroids and avoiding offending agents.
